# Prevention of D-GalN/LPS-induced ALI by 18β-glycyrrhetinic acid through PXR-mediated inhibition of autophagy degradation

**DOI:** 10.1038/s41419-021-03768-8

**Published:** 2021-05-13

**Authors:** Shouyan Wu, Henglei Lu, Wenjie Wang, Luyao Song, Meng Liu, Yuhan Cao, Xinming Qi, Jianhua Sun, Likun Gong

**Affiliations:** 1 State Key Laboratory of Drug Research, Shanghai Institute of Materia Medica, Chinese Academy of Sciences, Shanghai, 201203 China; 2 University of Chinese Academy of Sciences, Beijing, 100049 China; 3 Shanghai University of Traditional Chinese Medicine, Shanghai, 201203 China; 4 Department of Pharmacology, Fudan University, Shanghai, 201203 China; 5 Zhongshan Branch, the Institute of Drug Discovery and Development, Chinese Academy of Sciences, Zhongshan, China

**Keywords:** Metabolic disorders, Immunopathogenesis

## Abstract

Acute liver injury (ALI) has multiple causes and results in liver dysfunction. Severe or persistent liver injury eventually leads to liver failure and even death. Pregnane X receptor (PXR)-null mice present more severe liver damage and lower rates of autophagy. 18β-glycyrrhetinic acid (GA) has been proposed as a promising hepatoprotective agent. We hypothesized that GA significantly alleivates D-GalN/LPS-induced ALI, which involved in PXR-mediated autophagy and lysosome biogenesis. We found that GA can significantly decrease hepatocyte apoptosis and increase the hepatic autophagy marker LC3-B. Ad-mCherry-GFP-LC3 tandem fluorescence, RNA-seq and real-time PCR indicated that GA may stabilize autophagosomes and lysosomes and inhibit autophagosome–lysosome fusion. Simultaneously, GA markedly activates PXR, even reversing the D-GalN/LPS-induced reduction of PXR and its downstream genes. In contrast, GA has a weak protective effect in pharmacological inhibition of PXR and PXR-null mice, which significantly affected apoptosis- and autophagy-related genes. PXR knockout interferes with the stability of autophagosomes and lysosomes, preventing GA reducing the expression of lysosomal genes such as Cst B and TPP1, and suppressing autophagy flow. Therefore, we believe that GA increases autophagy by inhibiting autophagosome–lysosome fusion and blocked autophagy flux via activation of PXR. In conclusion, our results show that GA activates PXR to regulate autophagy and lysosome biogenesis, represented by inhibiting autophagosome–lysosome fusion and stabilization of lysosome. These results identify a new mechanism by which GA-dependent PXR activation reduces D-GalN/LPS-induced acute liver injury.

## Introduction

Acute liver injury (ALI) results in the massive death or loss-of-function of hepatocytes, and severe or persistent liver injury eventually leads to liver failure. Acute liver failure is a serious outcome of acute liver injury and is rare but life-threatening. In developed countries, fewer than 10 cases per million people occur each year, but the mortality rate is as high as 40–80%^1,2^. The rarity, severity, and heterogeneity of acute liver failure have led to very limited treatment options. Acute liver injury and acute liver failure are continuous and progressive pathophysiological processes, and acute liver injury is an early manifestation of acute liver failure. Therefore, intervention upon acute liver injury is key to preventing and treating acute liver failure. Licorice is a widely used herb in southern Europe and parts of Asia that is commonly found in medicine and confectionery^3^. Since Japanese scholars confirmed that licorice preparations can be used to treat patients with chronic hepatitis in a double-blind study in 1977^4^, an increasing number of studies has confirmed that licorice preparations have significant antihepatic effects. 18β-Glycyrrhetinic acid (GA), as the main active metabolite of licorice, has been widely proven to have significant anti-inflammatory, antioxidant, and antiapoptotic effects^5^. However, its protective mechanism against D-GalN/LPS (D/L) -induced acute liver injury is unknown. In this article, we used the D/L-induced acute liver injury model to explore the hepatoprotective effect of GA and its potential mechanism. This article aims to explore the pathogenesis of acute liver injury and provide guidance for the clinical use of GA.

Autophagy (macroautophagy) is a cellular system that degrades unnecessary substances (including proteins and organelles) in cells for recycling^6^. It is essential for maintaining liver homeostasis under both healthy and pathological conditions. Under stress conditions, the cell membrane or plasma membrane invaginates, and encloses damaged organelles or other substances. Then under the action of the autophagy formation-related protein ATG, the membrane vesicles are extended to form autophagic vesicles^7^. Autophagic vesicles have a bilayer membrane structure, in response to actin or synapses, the outer membranes of the autophagic vesicles and secondary lysosomes fuse to form autophagosomes. The cytoplasmic components in the autophagosome inner membrane are transported into the lysosome, where they are degraded by hydrolases (such as cathepsins). Autophagy plays an important role in both cell death and cell survival. It has been reported that activating autophagy can significantly reduce GCDC-induced cholestatic liver injury in LO2 cells^8^. GA can activate autophagy by inhibiting the IRE1α-JNK/c-Jun signaling pathway and inhibit the proliferation of sarcoma cells^9^. There are also reports that inhibiting autophagy can alleviate fibrosis induced by carbon tetrachloride (CCl4)^10,11^, and reduce tumor cell migration and invasion^12^. Therefore, autophagy is considered to be a double-edged sword that can maintain the normal function of cells at low levels. However, autophagy is rapidly upregulated under stress conditions such as nutritional deficiencies. Excessive phagocytosis can lead to overwhelming degradation of mitochondria or other essential components of cells, leading to cell death^13–15^. Autophagy is a highly dynamic and multistep process. The accumulation of autophagosomes may be reflected in increased autophagy formation or hindered downstream processes of autophagy, such as low fusion efficiency or reduced lysosomal degradation. Therefore, autophagic flux is used to represent the dynamic process of autophagy and is the most accurate metric to evaluate the autophagy process. The literature confirms that increasing autophagic flux can significantly reduce a variety of liver injuries^16,17^, and small-molecule inhibitors or microRNAs can also inhibit autophagic flux and alleviate liver damage^18–21^.

Pregnane X receptor (PXR, also known as NR1I2) is a typical member of the nuclear receptor superfamily and is activated by endogenous and heterologous organisms. PXR is mainly involved in detoxification, metabolism, inflammation, apoptosis, and oxidative stress in a ligand-dependent manner. Pregnenolone 16α-carbonitrile (PCN), an effective agonist of PXR, can significantly mitigate Con A-induced immune liver damage^22^ and attenuate CCl4-induced liver fibrosis in mice^23^, and PXR knockout can significantly increase the extent of D/L-induced liver damage^24^. Studies have indicated that in hepatocytes with ammonia-induced PXR overexpression (or rifampicin-induced), LC3-B is downregulated and P62 is upregulated, but the opposite occurs in PXR-knockdown (PXR−) liver cells, which is due to the inhibition of P53/AMPKβ by PXR in ammonia-treated hepatocytes^25^. Interestingly, in D/L-treated animals, PXR knockdown can significantly reduce LC3-A, LC3-B, and Beclin-1 protein levels^24^, but the mechanism involved is not clear.

We found that D/L-treated rat and mouse livers have significantly reduced expression of genes downstream of PXR and that PXR has a significant liver protective effect in PXR-null mice treated with D/L. Autophagy regulates liver functions and maintains homeostasis in the liver^26^. In this study, we hypothesized that GA-mediated alleviation of D/L-induced liver injury is related to PXR-mediated regulation of autophagy. We found that GA can significantly stabilize autophagosomes and lysosomes, inhibit autophagosome–lysosome fusion, and increase autophagy. We also found that GA can significantly activate PXR and that PXR has a significant effect on autophagosome–lysosome fusion. Therefore, our findings highlight the role of PXR in autophagosome–lysosome fusion pathways and GA in preventing D/L-induced acute liver injury.

## Materials and methods

### Animals and experimental design

Male Sprague-Dawley rats and c57bl/6 mice were purchased from Shanghai Laboratory Animal Co. (Shanghai, China). The feeding conditions were in accordance with the specific pathogen-free (SPF)-grade requirements of the Institutional Ethics Committee of Shanghai Institute of Materia Medica. The relative humidity was 30–70%, the light/dark cycle was 12/12 h, and diet and drinking water were provided ad libitum. Animals were preadministered an intraperitoneal injection of GA or other agents as scheduled. The rat model of acute liver injury comprises a single intraperitoneal administration of 800 mg/kg D-GalN and 30 μg/kg LPS 24 h before the end of the experiment, whereas the mouse model is a single intraperitoneal administration of 300 mg/kg D-GalN and 10 μg/kg LPS 4 h before the end of the experiment. After the experiment, plasma was collected and centrifuged at 3000*g* for 8 min. One part of the liver was fixed, and another part was frozen at −80 °C until further use.

### Liver injury analysis

The levels of plasma liver enzymes were measured with Roche kits (ALT, AST kits) and Roche biochemical analyzers. Fresh liver tissue samples were fixed in 4% paraformaldehyde in PBS, embedded in paraffin and sectioned at a thickness of 4 μm for hematoxylin & eosin (H&E) staining, TUNEL staining (Cat. No. 12156792910, Roche, Germany) and immunochemistry (KIHC-5, Proteintech, Wuhan, China) according to standard procedures and kit manuals. Histological lesions were scored by a pathologist. We evaluated tissue damage using a semiquantitative method according to the literature^27^. For semiquantitative analyses, the necrotic area or lesions in the target region were scored as +1 for <25%, +2 for 25–50%, +3 for 50–75%, and +4 for >75%. In the immunochemistry assay, the antibodies, their dilutions and applications were as follows: anti-LC3A/B, 1:100 (Immunofluorescence, #12741, CST, Boston, USA), goat anti-rabbit IgG H&L (Alexa Fluor^®^ 488), 1:1000 (Immunofluorescence, ab150077, Abcam, Cambridge, UK), anti-Stx 17, 1:500 (Immunoprecipitation, 17815-1-AP, Proteintech, Wuhan, China), and anti-Vamp 8, 1:10000 (Western blot, ab76021, Abcam, Cambridge, UK).

### RNA isolation and qRT-PCR analysis of mRNA expression

From 25 ± 3 mg of liver tissue, total RNA was isolated by using TRIzol reagent (Invitrogen, CA, USA) and an UNlQ-10 RNA extraction column kit (B511361, Sangon Biotech, Shanghai, China). cDNA was then reverse transcribed from RNA by PrimeScript™ RT Master Mix (RR036A, Takara, Shiga, Japan), and quantitative real-time polymerase chain reactions (qRT-PCR) were analyzed on an ABI 7500 Fast system (ABI, CA, USA) using Hieff^®^ qPCR SYBR Green Master Mix (11202ES03, Yeasen, Shanghai, China). The primer sequences are listed in Supplementary Table 1. All results were normalized to GAPDH expression and calculated using the 2^-(ΔΔCt)^ method.

### Western blotting

Western blotting was performed on total proteins extracted from mouse livers, rat livers and cell lines. The samples were homogenized with radioimmunoprecipitation assay buffer (+1% phenylmethanesulfonyl fluoride) (Beyotime, Shanghai, China), sonicated, centrifuged, and quantificated with a Pierce^TM^ BCA protein assay kit (#23225, Thermo, MA, USA). An aliquot of the protein lysate was added to 5× SDS loading and incubated at 95 °C for 7 min. Equal amounts of total proteins were resolved by SDS-PAGE on a 10% gel and transferred to PVDF membranes. The membranes were incubated overnight at 4 °C with antibodies, as shown in Supplementary Table 2. Chemiluminescence of the protein bands was detected using ECL (JP001B250, Clinx, Shanghai, China) and an immunoblot detection system (Clinx, Shanghai, China), and the protein band densities were quantified by ImageQuant software (GE Healthcare, Hertfordshire, UK).

### PXR luciferase assay

The luciferase reporter expression plasmids pcDNA3.1-PXR and PGL3-CYP3A4-XREM were purchased from YouBio (Hunan, China), and the Renilla luciferase gene-containing plasmid pRL-SV40 was used for normalization of luciferase activity. We seeded HEK293T cells (ATCC, Virginia, USA) in 48-well plates for luciferase assay experiments and cultured them until they reached 80–90% confluence, after which transient transfection was conducted with Lipofectamine 2000 (Life Technologies, CA, USA) according to the instructions of the manufacturer. In the transactivation system, 200 ng of pcDNA3.1-PXR, 200 ng of PGL3-CYP3A4-XREM-Luc, and 50 ng of pRL-SV40 were transfected into the cells. After 6–8 h, the medium was changed to DMEM and incubated with different concentrations of GA for 24 h and 5 μM rifampicin as a positive control. Luciferase activity was measured using a dual-luciferase reporter assay system (Promega, Madison, WI, USA).

### Adenovirus-infected cells

LO2 cells were seeded into 12-well plates. When the cells reached 80% confluence, adenovirus Ad-mCherry-GFP-LC3 (MOI = 10, C3011, Beyotime, Shanghai, China) was added to the cells for 24 h. Next, culture medium containing the virus was removed and replaced with medium containing different concentrations of agents for 24 h, and cell growth and infection efficiency were observed.

### Statistical analysis

Data were statistically analyzed and graphed using GraphPad Prism software (version 5.03; GraphPad Software, Inc., CA, USA). All cell experiments were performed independently at least three times. The data are expressed as mean ± SD, and one-way ANOVA was used to analyze the significance of differences. *P* < 0.05 was considered statistically significant (^*^P < 0.05, ^**^P < 0.01, ^***^P < 0.001).

## Results

### GA pretreatment protects rat liver from D-GalN/LPS-induced acute liver injury in vivo and in vitro

**Fig. 1:**
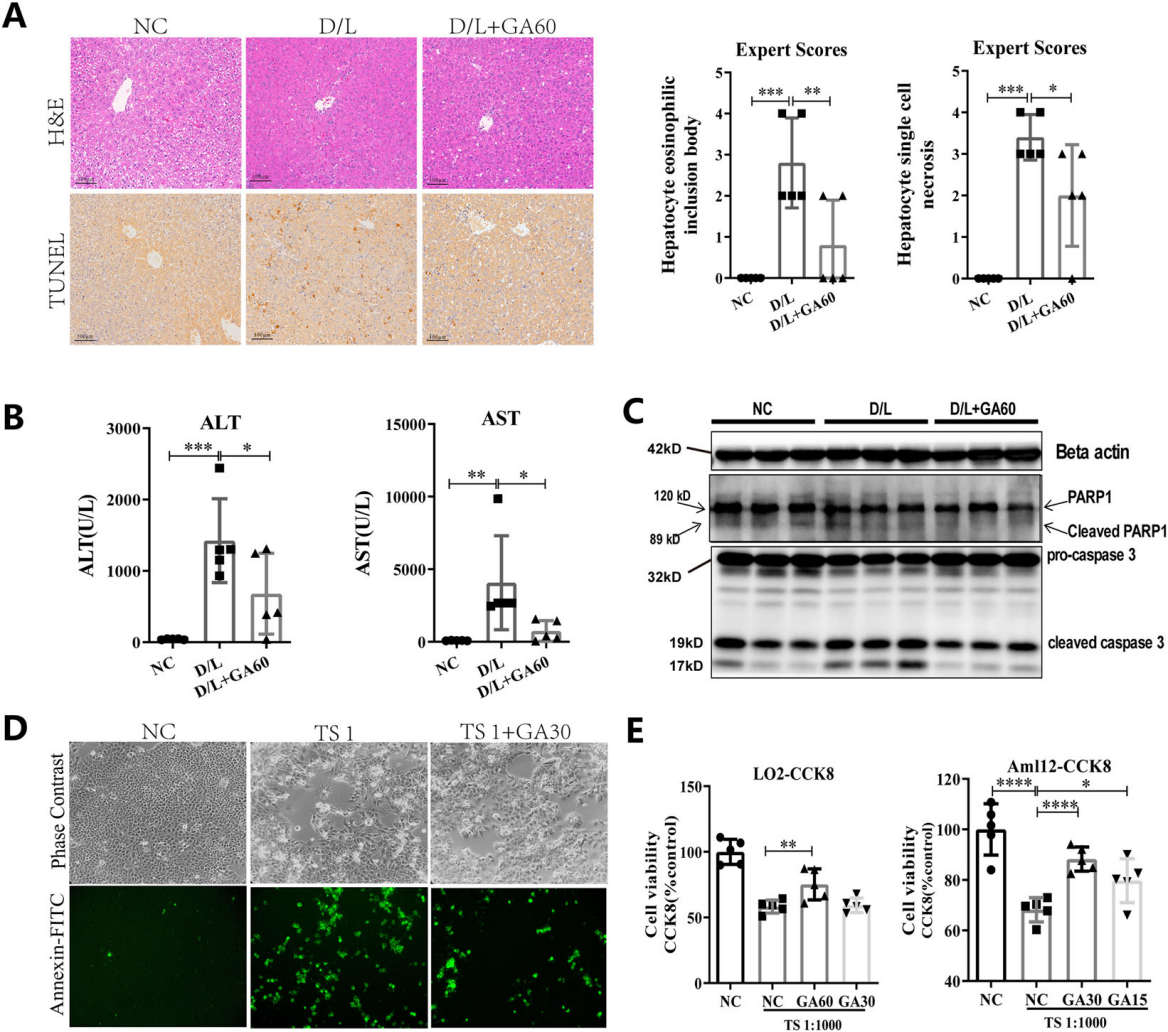
Effect of GA on acute liver injury in vivo and in vitro. SD rats were intraperitoneally administered GA (60 mg/kg) or vehicle for 5 days, and then treated with 800 mg/kg D-GalN plus 30 μg/kg LPS for 24 h (*n* = 5). H&E and TUNEL stained liver slices and corresponding histological scores (**A**), plasma liver enzymes ALT, AST (**B**), and hepatocyte apoptosis (**C**) in rat livers were detected. Cells were treated with TS (1 μl/ml, C0006S, Beyotime) and with or without GA (μM) for 24 h, the cell viability was measured by using FITC fluorescence in LO2 cells (**D**) and CCK-8 in LO2 and Aml 12 cells (**E**). Data represent three independent experiments. Data are shown as mean ± SD, statistical significance: ^*^P < 0.05, ^**^P < 0.01, ^***^P < 0.001, ^****^P < 0.0001.

To determine the hepatoprotective effect of GA, a D/L-induced acute liver injury model was established in SD rats pretreated with GA for 7 days. Liver plasma enzyme levels, histological analysis and TUNEL staining results (Fig. 1A, B) all show that D/L can significantly increase plasma alanine aminotransferase (ALT) and aspartate aminotransferase (AST) levels and pathological damage, and GA can effectively reverse these phenomena. Western blot results also confirmed that GA can effectively reverse D/L-induced apoptosis of rat hepatocytes (Fig. 1C). Subsequently, we examined the effect of GA on liver injury in vitro using TS-induced LO2 and Aml12 cell models. By using Annexin-FITC staining and the CCK-8 assay, we observed that GA reduced the number of TS-induced FITC-positive cells (Fig. 1D), and GA can significantly promote the cell viability of TS-induced LO2 and Aml12 cells (Fig. 1E). These data suggested that GA has a good hepatoprotective effect in vivo and in vitro.

### GA pretreatment can increase the accumulation of LC3-B and P62 in D-GalN/LPS-induced acute liver injury

**Fig. 2:**
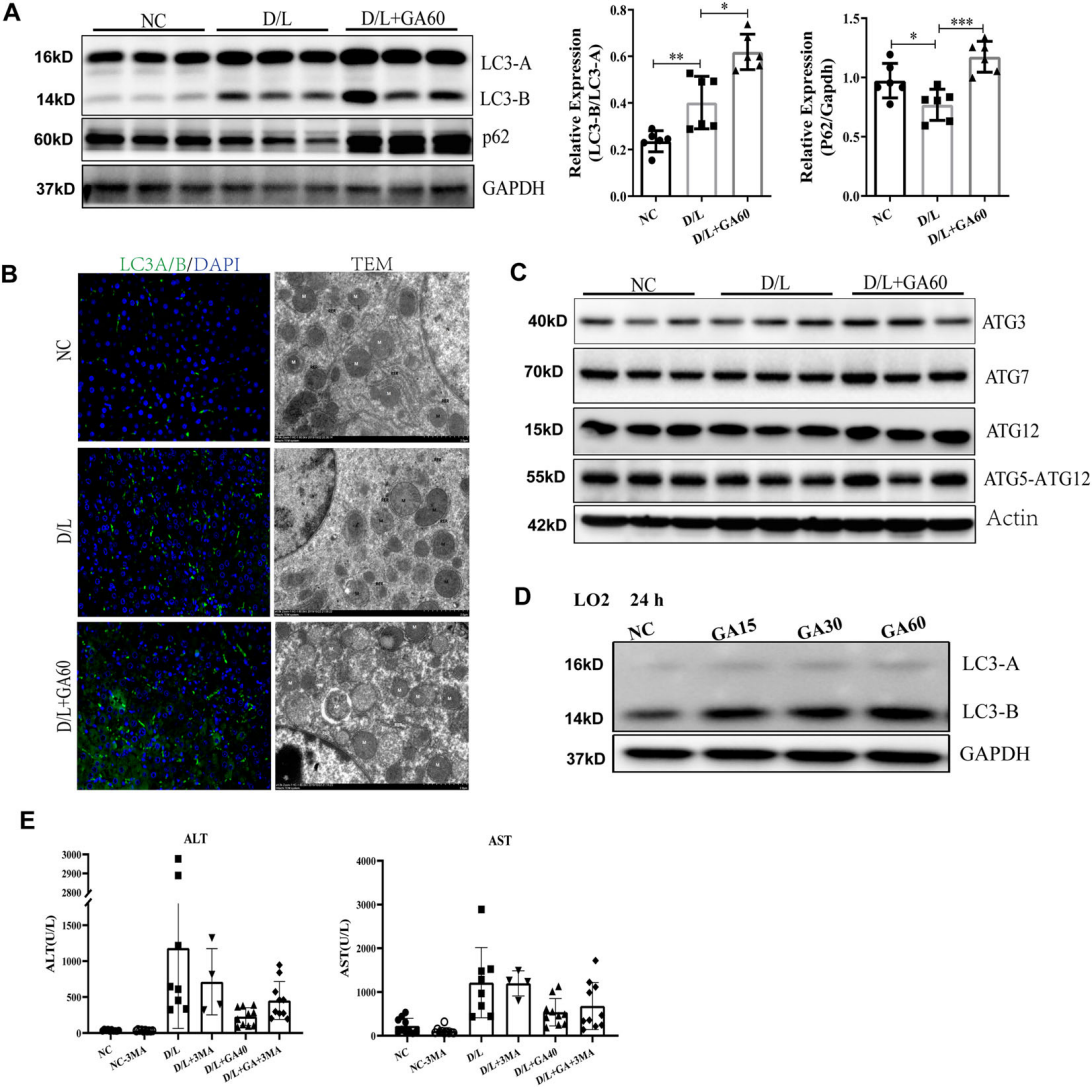
GA treatment causes the accumulation of LC3-B and P62. In rat liver tissue (*n* = 6), the levels of LC3-B and P62 (**A**) and autophagosome formation-related proteins (**C**) were detected by western blot, autophagosomes were detected by immunofluorescence staining and transmission electron microscopy (TEM) images (**B**). LO2 cells were treated with different concentrations of GA for 24 h and the level of LC3-B was examined by western blot (**D**). ALT and AST of C57 mice cotreated with D/L and with or without GA (40 mg/kg) or 3-MA (the autophage inhibitor, 30 mg/kg) were measured (*n* = 10) (**E**).

The role of autophagy in D/L-induced acute liver injury is not fully understood^28,29^. Immunoblot analysis and transmission electron microscopy (TEM) were performed in rats treated with GA and subjected to D/L. GA can upregulate the protein levels of the autophagy-related genes LC3-B and P62, and immunofluorescence also showed that GA significantly promoted LC3-B expression (Fig. 2A, B). In addition, TEM data confirmed that GA can significantly increase the number of autophagic vesicles (Fig. 2B). We evaluated the induction of autophagy and found no significant changes in either the Atg5-12 conjugate or the expression of two LC3 lipidation proteins (Atg3 and Atg7) (Fig. 2C). GA also increased LC3-B levels in LO2 cells in a dose-dependent manner (Fig. 2D). These data indicated that GA promoted the accumulation of LC3-B and P62, which is not due to increased autophagy formation. In order to confirm that the hepatoprotective effect of GA is closely related to autophagy, we used upstream autophagy inhibitors (3-MA) to block the initiation of autophagy. It was found that under the condition of no damage, 3-MA did not aggravate liver damage, indicating that it had no liver toxicity under the current/normal conditions. But, D/L and 3-MA cotreatment was significantly increased the mortality of mice (60% mortality), indicating that inhibited autophagy can significantly aggravate liver damage. In addition, we found that GA (D/L + GA group) can significantly inhibit the increase of ALT and AST, and after 3-MA treatment (D/L + GA + 3-MA group), the liver-lowering enzyme effect of GA was significantly weakened (Fig. 2E). These data indicate that the protective effect of GA on acute liver injury is achieved by increasing autophagy.

### GA inhibits autophagy–lysosomal fusion and blocks autophagy flux

**Fig. 3:**
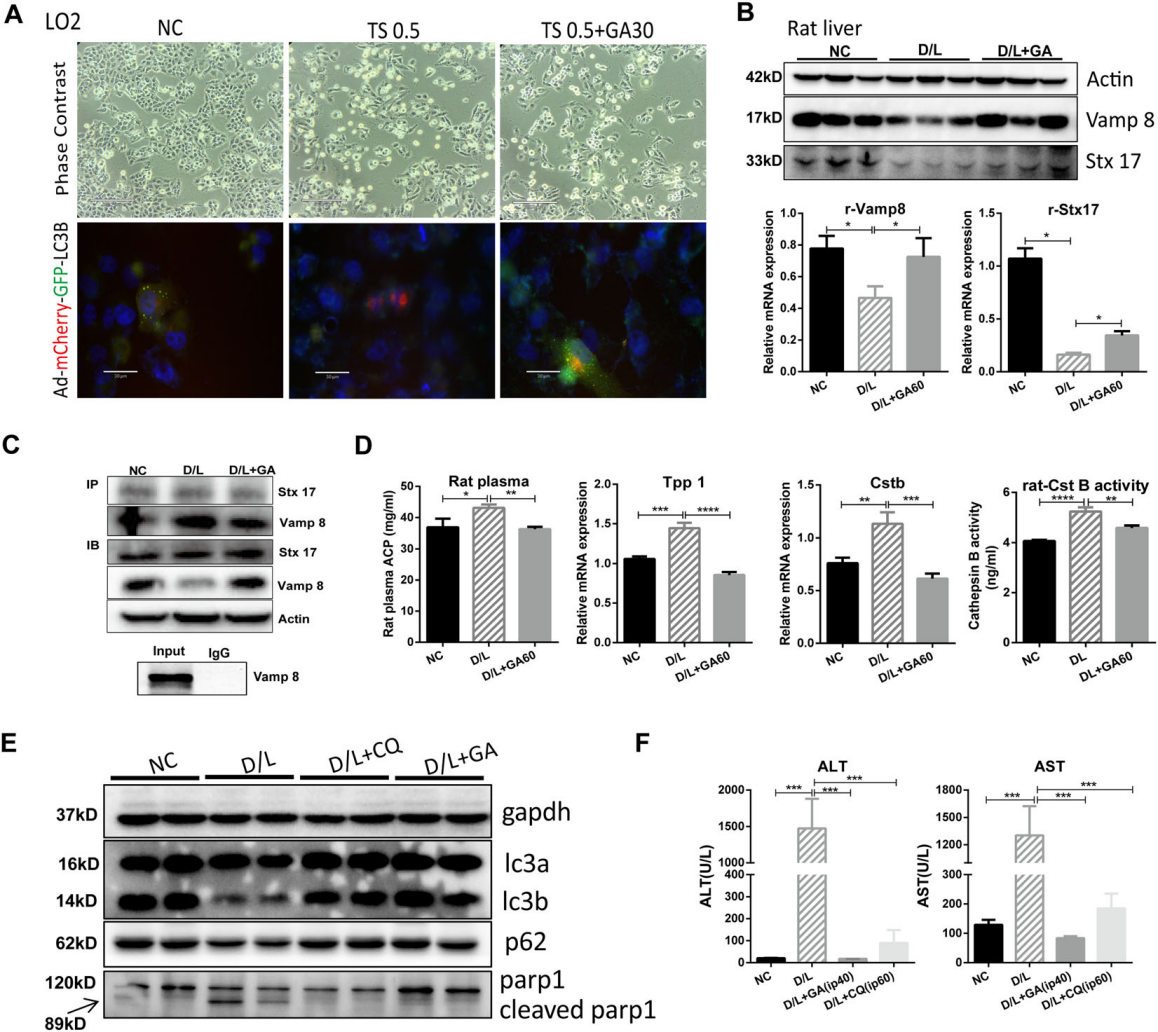
Effect of GA on autophagosome–lysosome fusion and degradation. LO2 cells were transfected with GFP-mCherry-LC3 for 24 h, and treated with TS (0.5 μl/ml) and/or GA (30 μM) for 24 h, the fluorescence images were captured by microscopy, scale bar = 30 μm (**A**). The lysosome and autophagosome markers Vamp 8 and Stx17 protein levels were detected by western blot and RT-PCR (**B**), their interactions were detected by co-immunoprecipitated (**C**) and effect of GA on lysosomal integrity were measured (**D**) in rat liver. C57bl/6 mice (*n* = 8) cotreated D/L with or without CQ (60 mg/kg) or GA (40 mg/kg) for 5 days (*n* = 8), and the expression of LC3-B, P62 and cleaved Parp1 (**E**), ALT and AST (**F**) were measured. Data are shown as mean ± SD, statistical significance: ^*^P < 0.05, ^**^P < 0.01, ^***^P < 0.001.

To determine whether GA exposure prevents autophagosome fusion with lysosomes, we transfected LO2 cells with a plasmid expressing mCherry-GFP-LC3 to monitor the autophagic flux status via fluorescence microscopy. We observed that red puncta were predominant in the TS-treated group, indicating that autophagosomes had fused with lysosomes. In the TS + GA cotreatment group, almost all the LC3-B dots presented yellow fluorescence, suggesting that GA significantly inhibited the fusion of autophagosomes with lysosomes (Fig. 3A).

Vamp 8 and Stx 17 are important proteins on the membranes of lysosomes and autophagosomes, respectively, and can be used to characterize the quantity and integrity of lysosomes and autophagosomes^30,31^. Our data showed that GA significantly increased the protein and mRNA levels of Vamp 8 and Stx 17 in rat liver tissues (Fig. 3B). Co-IP results also confirmed that GA can mitigate the interaction between Vamp 8 and Stx 17 (Fig. 3C). These data indicated that GA inhibits autophagosome–lysosome fusion and blocks autophagic flux. Lysosomes play an important role in autophagy by recycling organelles and long-lived proteins. Our results confirmed that GA treatment can effectively reduce acid phosphatase (ACP, a lysosomal marker) and autophagolysosomal-related genes (Cst B and TPP1) (Fig. 3D). These results implied that GA can significantly inhibit autophagy-mediated degradation. To further clarify the role of GA in blocking autophagic flux, we used the lysosome inhibitor, chloroquine (CQ) to inhibit autophagosome–lysosome fusion, which increases the pH of lysosomes, blocks the degradation of autophagosomes and subsequently determines LC3-B turnover. The accumulation of LC3-B and P62 caused by GA was comparable to that caused by CQ (Fig. 3E), which suggests that GA blocks autophagy as efficiently as does CQ. GA is similar to CQ in reducing liver plasma enzymes and reducing liver damage (Fig. 3E, F). These results suggest that the accumulation of LC3-B and p62 caused by GA is due to inhibition of autophagic flux.

### Transcriptome evidence reveals that GA can modulate autophagy and lysosome function

**Fig. 4:**
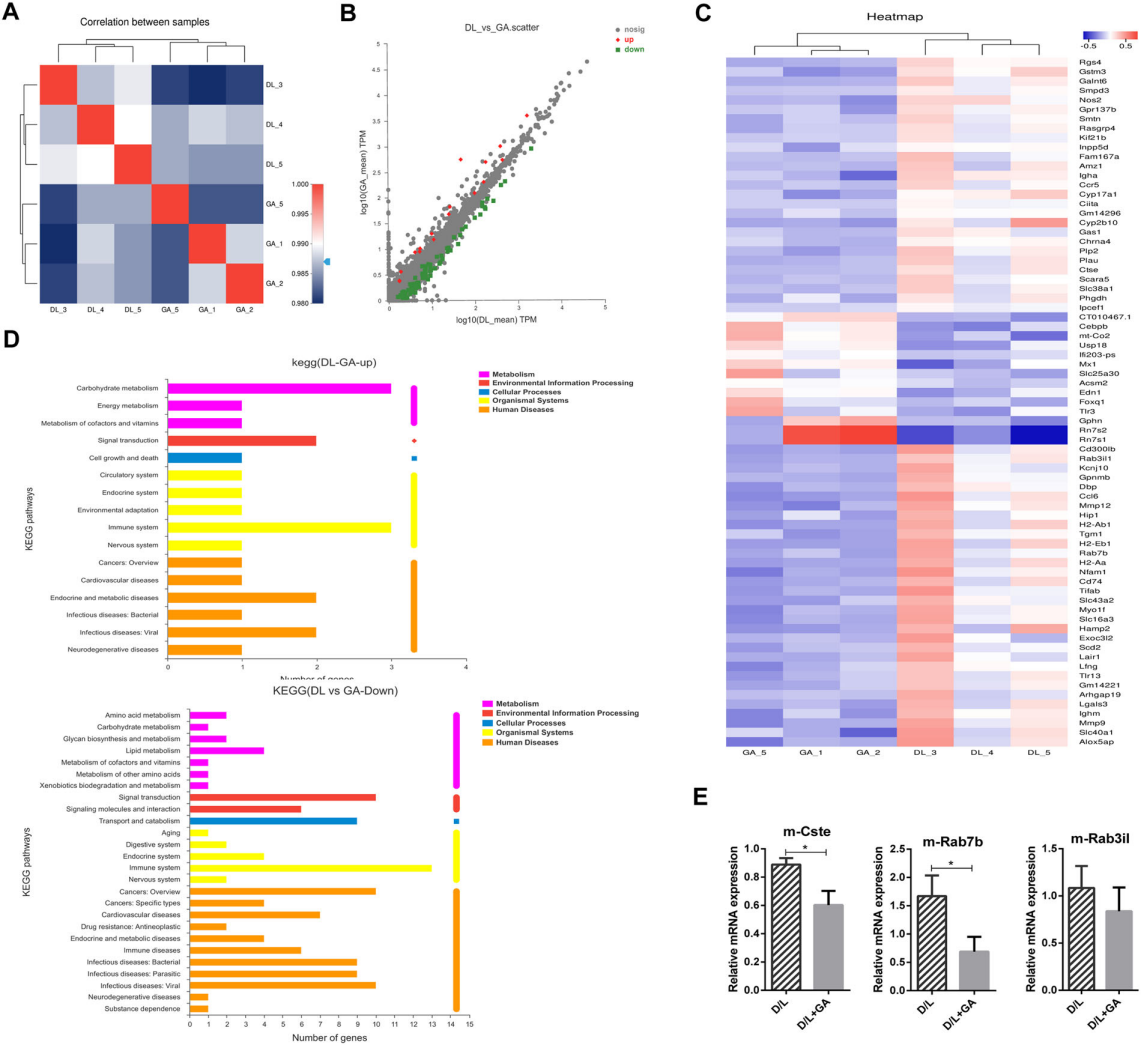
RNA-seq analysis of liver tissues from D/L- and/or GA-treated mice. Eight-week-old male mice were treated with or without GA for 5 days, and then treated with 300 mg/kg D-GalN plus 10 μg/kg LPS for 4 h (*n* = 3). Pearson’s correlation matrix of liver samples (**A**). Volcano plots (**B**) and Heat map (**C**) were used to depict the differential expression of liver genes. KEGG pathway analysis (**D**). Liver lysates from D/L- and/or GA-treated mice were used for the detection of autophagolysosomal SDEGs (Cst E, Rab7b, and Rab3il) by RT-PCR analysis (**E**). Statistical significance relative to the D/L group: ^*^P < 0.05.

To further confirm the above conclusions, we used high-throughput RNA-seq technology to assess the transcriptomes of livers from D/L-treated and D/L + GA-cotreated mice (*n* = 3, the data were analyzed on the free online platform of the Majorbio Cloud Platform (www.majorbio.com)). Heat map analysis showed close correlation between biological replicate samples (Fig. 4A) and significantly differentially expressed genes (SDEGs) between D/L and D/L + GA-cotreated mice as shown in Fig. 4B, C. KEGG pathway enrichment analysis of SDEGs showed that the upregulated SDEGs were mainly enriched in cell proliferation, death, and metabolism, while the downregulated genes were enriched in the regulation of transport, catabolism and metabolism (Fig. 4D). Importantly, we found that many more SDEGs are closely related to autophagy and lysosomes, such as Lyst, Rab3il1, Ctse, Rab7b, and Cebp/β (Fig. 4C). To validate the expression results generated by RNA-seq, we determined the mRNA levels of autophagolysosomal SDEGs (Cts e, Rab7b, and Rab3il1) by qRT-PCR, and they were all downregulated in D/L + GA-cotreated mice compared to D/L mice (Fig. 4E). RNA-seq data confirmed once again that GA can alleviate liver injury by regulating autophagy and lysosome function.

### GA alleviates liver injury by activating PXR

**Fig. 5:**
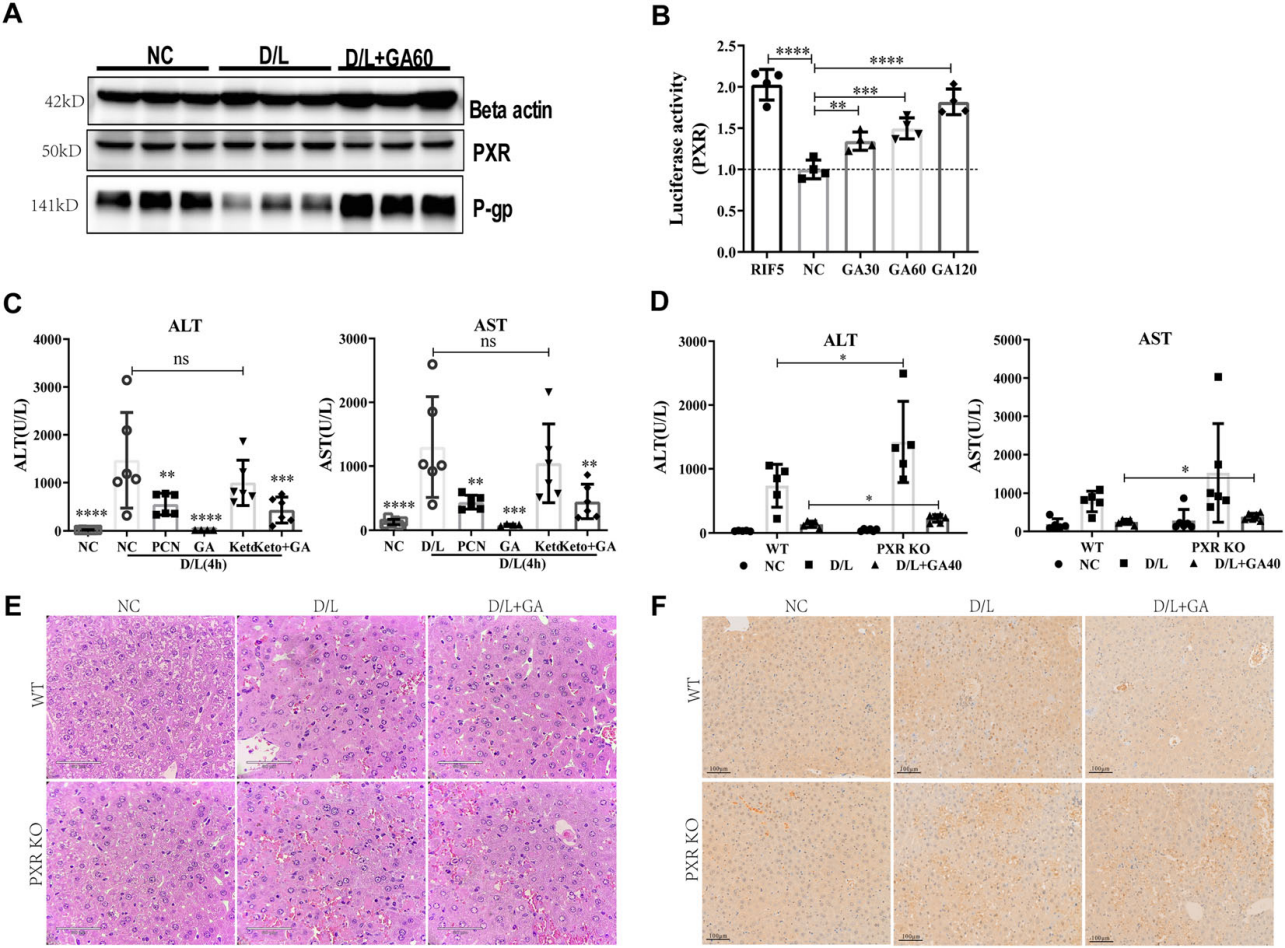
Interfering with PXR can effectively reduce the hepatoprotection of GA. Western blotting was used to determine the protein levels of PXR and its downstream effector P-gp in SD rats (**A**). The dual-luciferase reporter assay showed activation of PXR by GA in HEK293T cells and rifampicin was used as a positive control, data represent three independent experiments (**B**). Mice ALT and AST levels were measured in PXR pharmacological intervention (**C**) and genetic knockout (**D**). Liver sections were subjected to the indicated staining as H&E staining (**E**) and TUNEL staining (**F**). Statistical significance: ^*^P < 0.05, ^**^P < 0.01, ^***^P < 0.001, ^****^P < 0.0001.

There are 73 SDEGs in the aforementioned RNA sequencing (fold change ≥2). Gene ontology analysis showed that, in addition to autophagy, these genes can be broadly divided into several clusters, including inflammation and drug metabolism and transport clusters. In addition, upstream analysis with IPA found that PXR binds to the promoter of 15/73 genes in mouse liver (Supplementary Table 3). PXR (also known as NR1I2) plays an integral role in the homeostasis of liver pathology and physiology. D/L caused more serious hepatocyte apoptosis and liver damage in PXR-null mice, which have lower expression of LC3-A, LC3-B and Beclin-1^24^. We speculated that GA exerts liver protection by regulating PXR. We found that GA can significantly increase the protein expression of P-gp (which is downstream of PXR) in SD rat liver (Fig. 5A), the mRNA levels of PXR and its downstream genes are shown in Supplementary Fig. 1, and a luciferase assay confirmed that GA can activate PXR in vitro in a dose-dependent manner in vitro (Fig. 5B). These data indicated that GA has a significant activation effect on PXR. We used specific agents in mice to regulate PXR signaling and found that the PXR agonist pregnenolone (PCN) can significantly reduce D/L-induced increases in liver enzyme levels, while the PXR antagonist ketoconazole has no protective effect on D/L-induced liver injury (Fig. 5C). To explore the role of PXR more intuitively, we used PXR-null mice (genotyping results shown in Supplementary Fig. 2) to evaluate the protective effect of GA on liver injury. A lack of PXR resulted in a significantly higher ALT/AST, more serious pathological damage and increased hepatocyte apoptosis (more TUNEL-positive cells) after D/L treatment. GA cotreatment with D/L in PXR-null mice resulted in weaker reductions in ALT/AST, pathological damage (scores from pathologists listed in Supplementary Fig. 3) and hepatocyte apoptosis than those in D/L + GA-cotreated wild-type mice (Fig. 5D–F).

### PXR interferes with autophagy, which is closely related to the activity of GA in alleviating acute liver injury

**Fig. 6:**
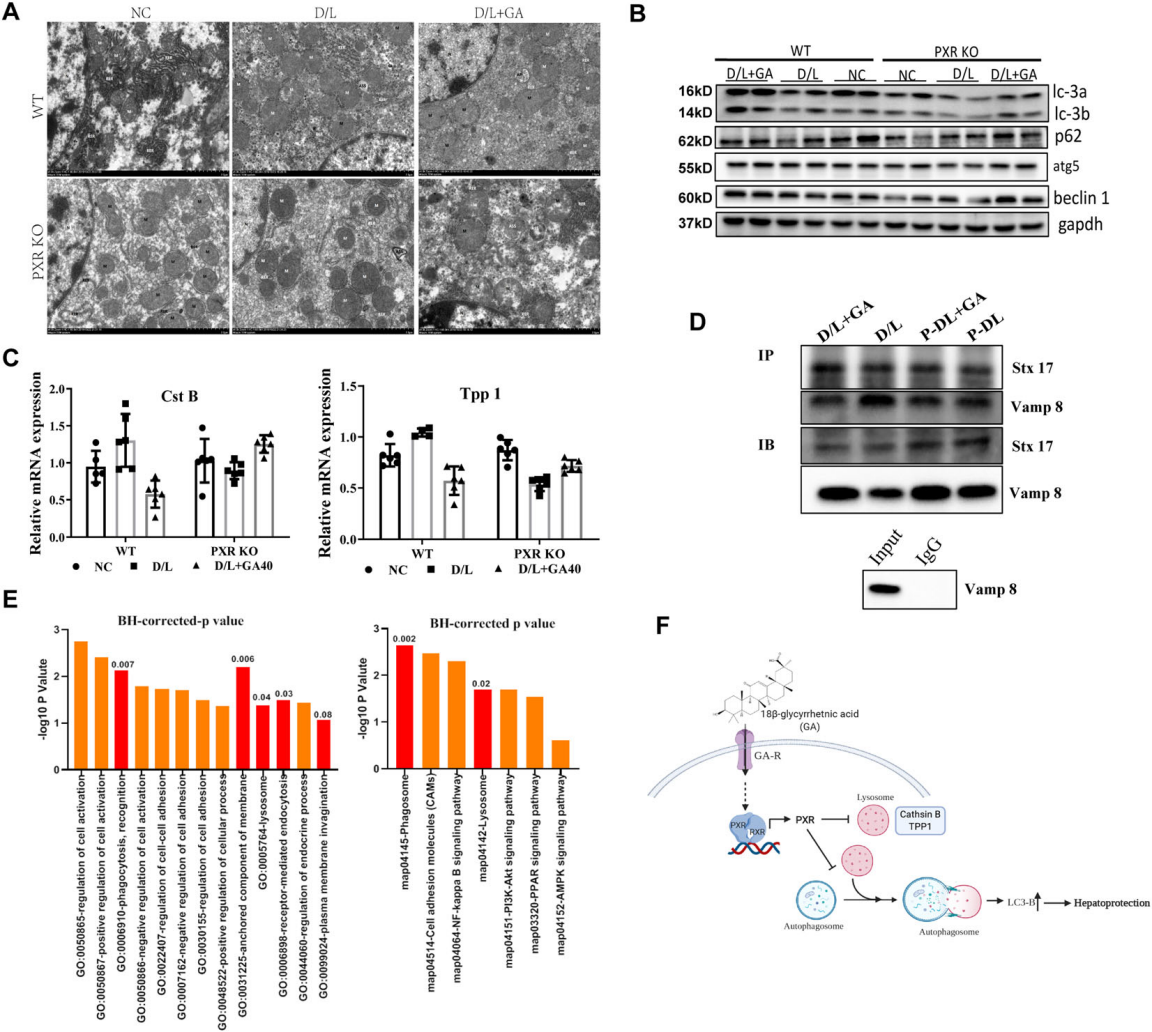
Knockout of PXR interferes with the autophagy regulation of GA in acute liver injury. Wild-type (WT) and PXR-null mice were treated with or without GA (40 mg/kg) for 5 consecutive days, and D/L was intraperitoneally administered at 4 h before the mice were sacrificed. **A** Transmission electron micrograph of liver sections. **B** The hepatic protein levels of autophagy-related biomarkers, and **C** the mRNA levels of autophagolysosomal-related genes (Cst B and TPP1) in mice were determined. **D** Co-IP of Vamp 8 and Stx17 in D/L- and/or GA-treated WT and PXR-null mice. IB, immunoblot. **E** Gene Ontology and KEGG enrichment analysis of SDEGs meeting a predefined threshold with a BH-corrected *P*-value < 0.05 in WT and PXR-null mice. **F** Pattern diagram of GA regulating autophagy through PXR.

Upon further exploring the regulatory effect of PXR on autophagy, we first found that PCN can significantly increase LC3-B and P62 levels but that ketoconazole has no effect (as shown in Supplementary Fig. 4), which indicated that PXR can regulate autophagy. TEM confirmed that the autophagosomes in the GA-treated group were significantly reduced in PXR-null mice compared to wild-type mice (Fig. 6A). Western blot also showed that in PXR-null mice, LC3-B and P62 levels in the GA-treated group were significantly reduced, and PXR knockout did not affect autophagy formation (Fig. 6B). Subsequently, we measured genes related to autophagosome and lysosomes (Cst B, TPP1) and the protein interaction between Vamp8 and Stx17. The results showed that PXR knockout can interfere with the stability of autophagosomes and lysosomes, prevent GA from reducing the expression of lysosomal genes such as CstB and TPP1 (Fig. 6C), and inhibit autophagosome–lysosome fusion (Fig. 6D). Furthermore, we used untreated wild-type and PXR-null mice to perform RNA-seq analysis. The GO and KEGG analyses of differentially expressed genes (heat maps and gene expression are shown in Supplementary Fig. 5) revealed enrichment in functions and processes such as phagocytosis, membrane invagination, and lysosomes (Fig. 6E), indicating that PXR can regulate autophagy and lysosome activity. These data confirmed that GA blocks autophagic flow, reverses D/L-induced liver injury, and is regulated by PXR.

## Discussion

Acute liver injury is a serious threat to human health, and its rapid disease course and complex etiology make its treatment more difficult. Although previous studies have found that autophagy is involved in the pathogenesis of acute liver injury and provide new directions for the prevention and treatment of liver diseases, different liver injuries can have different or even opposing autophagic effects. Moderate levels of autophagy can maintain cell metabolism and cell homeostasis, whereas excessive autophagy may cause cell death, which is not conducive to the survival of the organism.

Licorice root has been used in Chinese traditional medicine for thousands of years to reduce liver injuries associated with a number of clinical disorders including chronic hepatitis C infection^32^. After thousands of years of practice, licorice has proven to have detoxification, hepatoprotection, anti-inflammatory, antioxidation, immune regulation, antiviral, and even anticancer effects^33^. Currently, licorice is also widely used as a sweetener, food additive, and in cosmetics and has been approved by the US FDA as a food supplement^34^. Most Chinese medicine formulations approved by the China Food and Drug Administration use licorice as the main ingredient^35^. Licorice and natural products in licorice are also clinically used in Japan and Europe, mainly for the treatment of chronic viral hepatitis. The main component of licorice-isolated pentacyclic triterpenoids is glycyrrhizic acid (GL). GL is hydrolyzed by intestinal bacteria into GA, which is the main potent bioactive compound of GL in the body. GA has been used for many years in the treatment and prevention of hepatitis, chronic bronchitis, tumor growth and immunological disorders^36^, and GA has also been used as a potential therapy for alpha-naphthyl isothiocyanate (ANIT)-induced and carbon tetrachloride (CCl4)-induced liver injury. Mechanistically, GA induces the expression and transcriptional activation of Nrf2 and PPARγ and then inhibits NF-κB to further inhibit inflammation, and GA induces SOD/CAT and GSH-Px activities to resist cell oxidative stress. It has also been reported that GA can effectively mitigate D/L-induced acute liver injury, which is attributed to its anti-inflammatory and antiapoptotic effects^37,38^. The precise cellular and molecular mechanisms responsible for the clinical benefits of GA have not been identified. Recent evidence suggests that modulating autophagy alleviates liver damage in a mouse model of fulminant hepatic failure^39^. Our results show that GA can significantly reduce the increase in liver plasma enzymes ALT and AST induced by D/L. We used H&E and TUNEL to detect liver pathology, and also showed that GA has a good effect on reducing eosinophils, hepatocyte necrosis, and cell death. At the molecular level, WB results also showed that the cell death indicators cleaved caspase-3 and Parp-1 were significantly inhibited after D/L treatment, indicating that GA has a significant alleviating effect on D/L-induced liver injury. In vitro, we also obtained similar effect in vivo (Fig. 1).

Autophagy, as a highly conserved process in eukaryotes, participates in regulating whole-organism metabolism and maintaining cell homeostasis. We hypothesized that GA ameliorates D/L-induced acute liver injury due to its regulation of autophagy. Our results showed that LC3-B increased in the D/L group and further increased in the D/L + GA group (Fig. 2A, B). This may be due to the stress signal inducing autophagy when the liver is damaged (D/L group). Increased autophagy can maintain cell homeostasis and has a hepatoprotective effect (D/L + GA group). To further confirm the effect of autophagy on liver injury, we used the specific autophagy inhibitor 3-MA to treat D/L-induced mice (Fig. 2E). The results showed that the 3-MA + D/L group showed a large number of deaths (60% mortality). At the same time, to prove that liver damage is not caused by the toxicity of 3-MA, we used 3-MA to treat normal mice (that is, NC-3-MA) and found that 3-MA does not increase liver plasma enzymes, indicating that inhibition of autophagy can significantly aggravate liver damage in mice. When treated with GA (that is, the D/L + 3-MA + GA group), autophagy markers increased significantly, mortality decreased rapidly, and liver enzymes were significantly lower than those in the D/L group compared to the D/L + 3-MA group. These data indicate that GA has a hepatoprotective effect by reversing 3-MA-mediated autophagy inhibition. Autophagy is essential for maintaining cell survival, with autophagic flux monitoring considered one of the most effective ways to evaluate autophagy activation or inhibition^40^, this is achieved by observing autophagosome–lysosome fusion and lysosomal enzyme activity. To explore which processing of autophagy GA acting on, we monitored autophagic flux. Ad-mCherry-GFP-LC3 tandem fluorescent-tagged LC3B can be used to visualize the transition from neutral autophagosomes to acidic autolysosomes^41^. The fluorescence of GFP is quenched in the acidic compartment, while the fluorescence of mCherry is relatively stable in lysosomes. mCherry-GFP-LC3 emits both green and red fluorescence signals when the protein localizes to autophagosomes, which is often shown as yellow signals in merged pictures. Conversely, the fluorescence of GFP is quenched in autolysosomes, making autolysosomes appear red. In our results, compared with that of the NC group, the green fluorescence of the damaged cell group (TS group) was quenched and appeared red, implying that the protein at this time was in the lysosome (Fig. 3A). These data indicate that GA inhibited autophagy–lysosomal fusion and blocked autophagic flux. Soluble *N*-ethylmaleimide-sensitive factor attachment protein receptors (SNAREs) are the most important synaptic proteins in autophagosome-lysosome fusion. It has been reported that syntaxin 17 (Stx17) and vesicle-associated membrane protein 8 (Vamp8), which are markers of autophagosomes and lysosomes, respectively, regulate this process^42,43^. Stx17 is located in the outer membrane of intact autophagosomes, but not in the unsealed intermediate structure (separation membrane), which prevents the lysosome from fusing with the separation membrane. Stx17 has a unique C-terminal hairpin structure, which can interact with the lysosomal SNARE Vamp 8 to promote the fusion of autophagosomes and lysosomes^43^. Our results also show that GA can effectively reverse the D/L-induced decrease in the protein and mRNA levels of Stx 17 and Vamp 8 (Fig. 3B). This indicates that GA can stabilize autophagosome and lysosome membranes, which is consistent with the reduction in lysosomal enzyme leakage (Figs. 3D and 4C–E). Lysosomes play an important role in autophagy, they contain hydrolytic enzymes, such as acid phosphatase (ACP), cathepsin B (Cst B), and tripeptidyl-peptidase 1 (TPP1), which can effectively degrade damaged intracellular organelles and excess protein. Generally, intracellular Cst B is localized in lysosomes. Disruption of the lysosomes results in the release of Cst B into the cytosol, thereby causing the release of ROS and cytochrome c, and causing cell damage^44^. It has been reported that inactivation of Cst B may have a potential therapeutic effect in treating liver diseases^45,46^. To more intuitively explore the effect of GA blocking autophagic flux on liver injury, we selected CQ as a reference (Fig. 3F). CQ is an alkalinizing lysosomatropic drug that accumulates in lysosomes, where it increases pH and deacidification, impairs lysosomal enzymatic function, and blocks autophagic flux^47^. Compared with the D/L group, GA and CQ significantly and effectively alleviated liver damage. This shows that the inhibition of autophagic flux is beneficial to the liver, and the effect of GA is significantly better than that of CQ.

PXR, a nuclear transcription factor encoded by the nuclear receptor subfamily gene NR1I2, is involved in regulating the metabolism and clearance of exogenous toxic substances. Previous reports indicate that PXR is involved in regulating liver homeostasis depending on the expression of PXR-regulated phase I and II metabolic enzymes and drug transporters^48^, as well as in inflammation^49^, oxidative stress^50^, and apoptosis. Therefore, PXR may be a promising target to prevent and treat liver disease based on its transcriptional regulation in inflammation, liver injury, and homeostasis maintenance. A few studies have surmised that the effects of PXR on D/L-induced liver injury are related to its intervention in autophagy, but the specific mechanism is still unclear^24^. To determine whether and how PXR regulates autophagy in the scope of liver disease, we analyzed the expression and regulation of PXR in liver tissues with D/L-induced acute liver injury. The results showed that the genes downstream of PXR were significantly downregulated in D/L-induced liver injury, but the protein levels of PXR did not change significantly (Fig. 5). Based on this, we assume that PXR is involved in the occurrence or progression of liver injury. By using the PXR-specific agonist PCN and the inhibitor ketoconazole to intervene in autophagy in D/L-treated mice, we observed that PCN elicits a response similar to that of GA, which can significantly reduce D/L-induced liver enzymes, reduce the expression of the apoptosis-related gene PARP1 and increase the levels of the autophagy indexes LC3-B and P62. By contrast, the inhibitor ketoconazole has the opposite effect as PCN. Activation of PXR has been shown to have hepatoprotective effects in various liver injuries, including CCl4-induced and bile duct ligation-induced liver fibrogenesis^23,51^, ischemia-reperfusion liver injury^52^, primary biliary cirrhosis^53^ and other chronic liver diseases, and ANIT-, D/L-induced acute liver injury^54^. Mechanistically, it is believed that activating PXR can upregulate phase I enzymes, modulate hepatic drug metabolism, and induce drug clearance pathways. Interestingly, PXR activation has been shown to enhance APAP-induced liver injury^55^, precisely because PXR activation promotes the expression of phase I enzymes. Therefore, PXR is a double-edged sword in acute liver injury. PXR-targeted therapies offer the potential to be tailored to specific liver injuries, PXR antagonists for APAP-induced acute liver injury and PXR agonists for D/L-induced acute liver injury. It implicates PXR as a therapeutic target for liver injury, but they also caution against PXR activation by pharmaceutical drugs. Subsequently, we used PXR-null mice to further confirm the effect of PXR on hepatocyte autophagy and found that after PXR knockout, lysosomal gene expression and autophagosome–lysosome fusion are not regulated by GA (Fig. 6). Overall, these findings prompted us to speculate that PXR may have a positive effect on the progression of liver injury, and this positive effect is mainly involved in autophagosome–lysosome fusion.

In summary, we provide the first evidence for the activation of PXR by GA and the mechanism by which GA regulates the autophagy signaling pathway to alleviate acute liver injury both in vitro and in vivo. Specifically, we used wild-type and PXR-null mice to confirm that GA can significantly reduce liver plasma enzymes and histopathological damage. GA mainly relies on PXR to stabilize lysosomes and autophagosomes and inhibit autophagosome–lysosome fusion, thereby diminishing autophagic flux, causing LC3-B accumulation, reducing cell death, and relieving liver damage (Fig. 6F).

## Supplementary information

Supplementary figure legend.

Supplementary Fig. 1 GA treatment increases the mRNA levels of PXR and its downstream genes in rat liver.

Supplementary Fig. 2 The pattern and identification of PXR-null mice.

Supplementary Fig. 3 Liver pathological injury score.

Supplementary Fig. 4 The regulatory effect of PXR on autophagy.

Supplementary Fig. 5 Significantly differentially expressed genes (SDEGs) between the WT and PXR-null groups.

Supplementary Table 1.

Supplementary Table 2.

Supplementary Table 3.
