# Supplementary material for: Prevention of D-GalN/LPS-induced ALI by 18β-glycyrrhetinic acid through PXR-mediated inhibition of autophagy degradation
Source: Cell Death Dis. 2021 May 13;12(5):480. doi: 10.1038/s41419-021-03768-8 (PMC8119493; doi:10.1038/s41419-021-03768-8)
Supplement: Supplementary file 1 — Supplementary figure legend. [file 41419_2021_3768_MOESM1_ESM.docx]

**Supplementary Fig.1 GA treatment increases the mRNA levels of PXR and its downstream genes in rat liver.** SD rats were intraperitoneally administered GA (60 mg/kg) for 5 consecutive days. Then, 800 mg/kg D-GalN and 30 μg/kg LPS were intraperitoneally administered at 24 h before the end of the experiment. RT-PCR verification of the expression of PXR and its downstream genes (Mrp2, P-gp, Mrp3) were performed. All data are presented as the means ± SD. the statistically significant. *P < 0.05, **P < 0.01, ***P < 0.001.

**Supplementary Fig. 2 The pattern and identification of PXR-null mice.**

1. Illustration of the principle of PXR knockout. The CRISPR/Cas9 system was used to make a 41 bp deletion in the third exon of PXR, causing a frameshift mutation; this was completed by the Genetics Center of the Animal Institute of Beijing Medical Academy. (B) Genotyping of WT and PXR-null mouse livers. (C) The protein and mRNA levels of the indicated genes were analyzed by real-time quantitative PCR in liver samples from WT and PXR-null mice.

**Supplementary Fig. 3 Liver pathological injury score.**

(A)Schematic diagram showing the treatment of WT and PXR-null mice with D/L in the presence or absence of GA. (B) Histological scores for liver sections from D/L-treated mice pretreated with vehicle or GA. Data are presented as the mean ± SD.

**Supplementary Fig. 4 The regulatory effect of PXR on autophagy.**

Mice were given PXR-specific agents or GA for 5 days. The hepatic protein level of the autophagy biomarker was determined.

**Supplementary Fig. 5 Significantly differentially expressed genes (SDEGs) between the WT and PXR-null groups.** (A) Heat map plot for SDEGs in WT (NC) and PXR-null (pNC) mice. (B) Expression patterns of PXR, its downstream genes and three autophagolysosomal SDEGs in the WT and PXR-null groups.
