# Supplementary figures and images for: Prevention of D-GalN/LPS-induced ALI by 18β-glycyrrhetinic acid through PXR-mediated inhibition of autophagy degradation
Source: Cell Death Dis. 2021 May 13;12(5):480. doi: 10.1038/s41419-021-03768-8 (PMC8119493; doi:10.1038/s41419-021-03768-8)

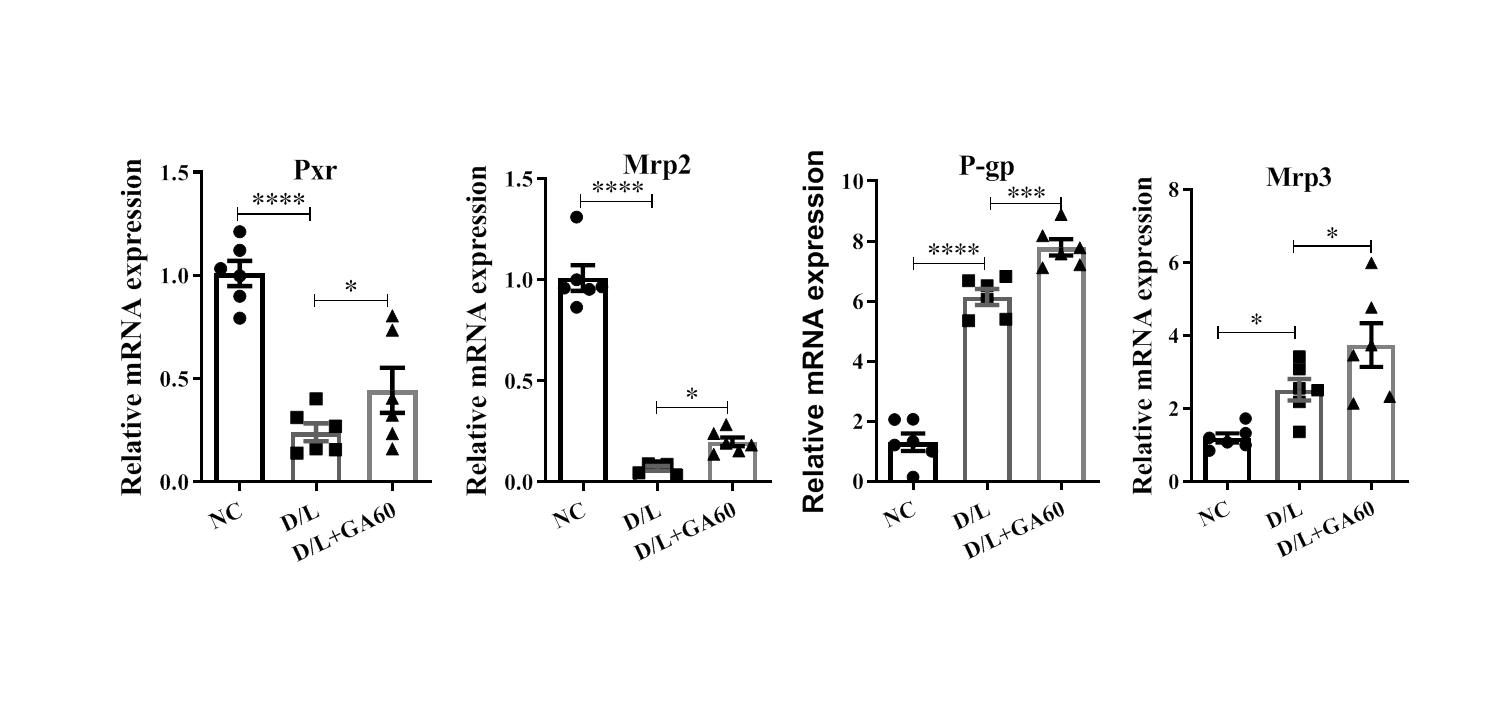

Supplement: Supplementary file 2 — Supplementary Fig. 1 GA treatment increases the mRNA levels of PXR and its downstream genes in rat liver. [file 41419_2021_3768_MOESM2_ESM.tif]

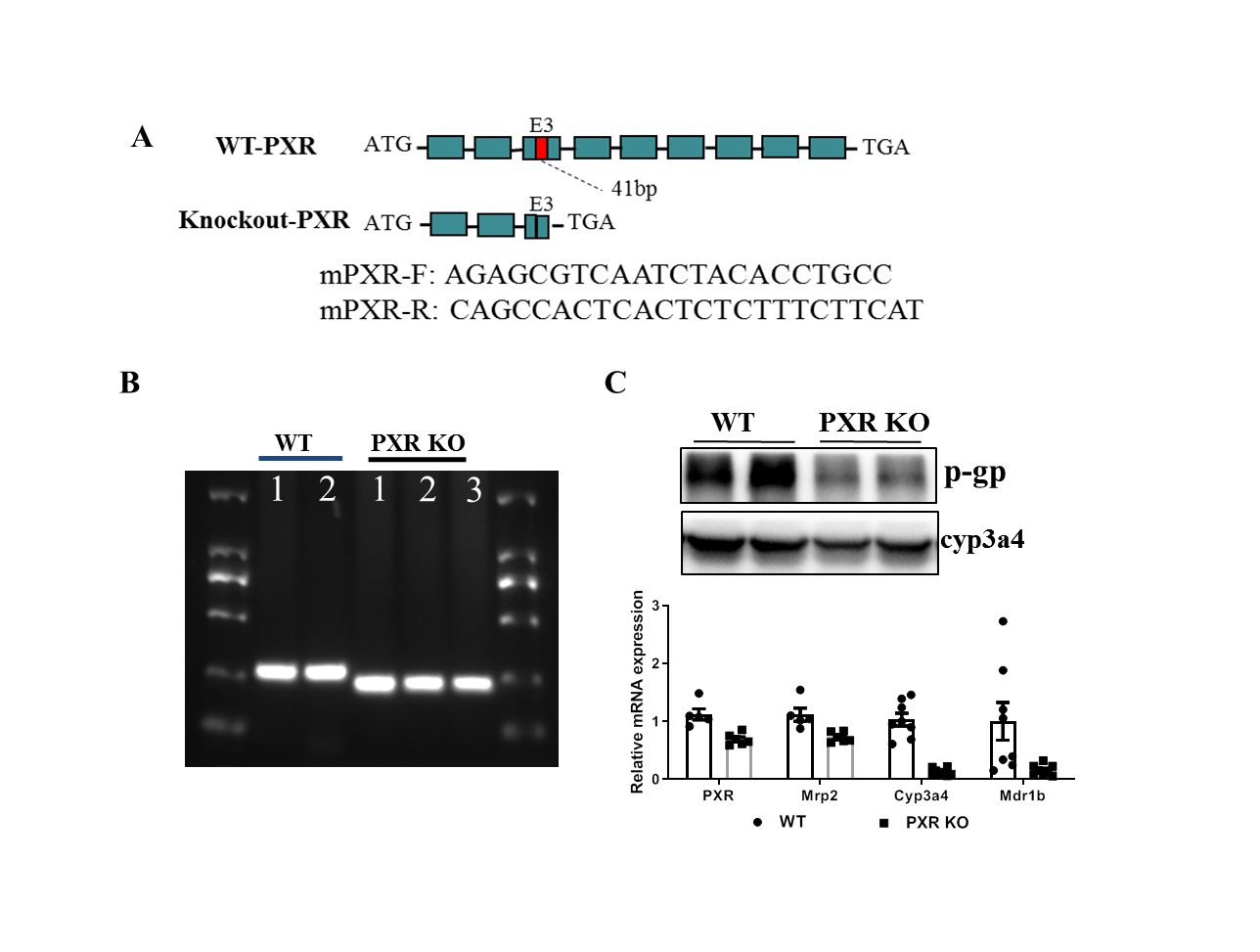

Supplement: Supplementary file 3 — Supplementary Fig. 2 The pattern and identification of PXR-null mice. [file 41419_2021_3768_MOESM3_ESM.tif]

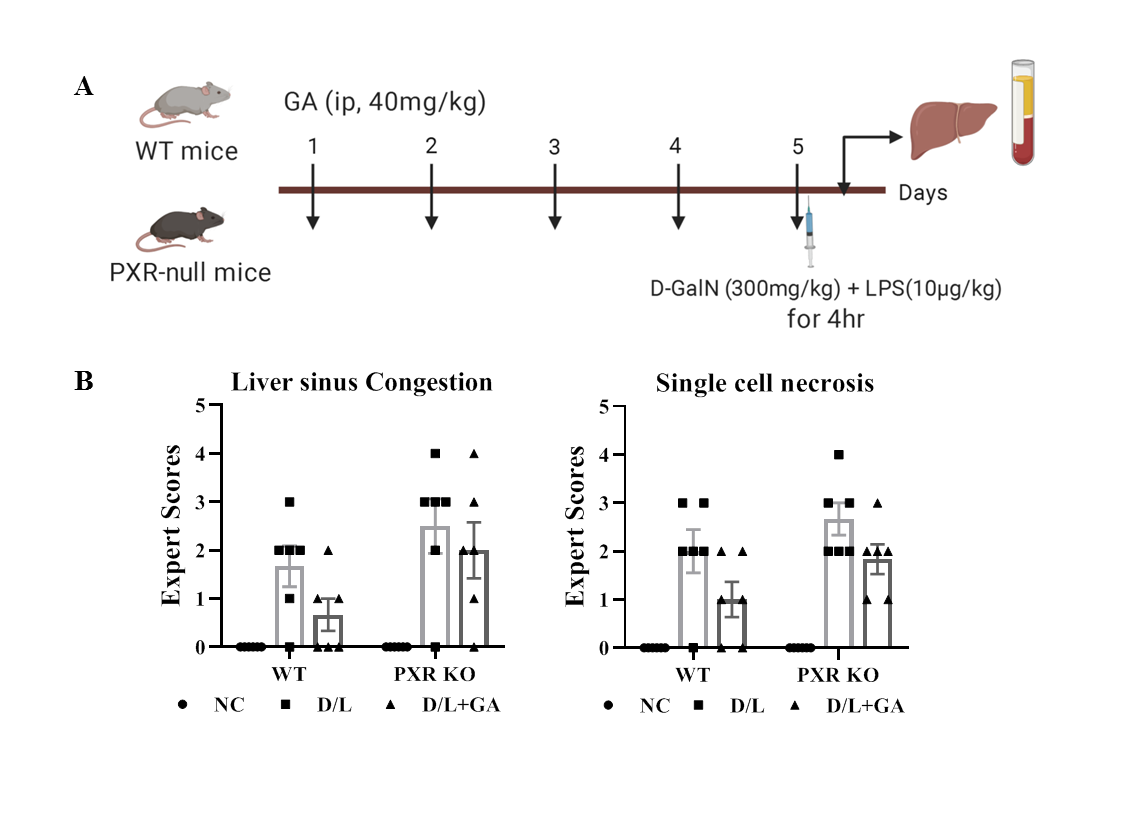

Supplement: Supplementary file 4 — Supplementary Fig. 3 Liver pathological injury score. [file 41419_2021_3768_MOESM4_ESM.tif]

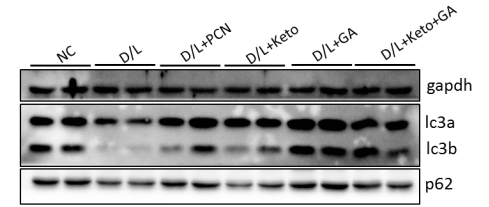

Supplement: Supplementary file 5 — Supplementary Fig. 4 The regulatory effect of PXR on autophagy. [file 41419_2021_3768_MOESM5_ESM.tif]

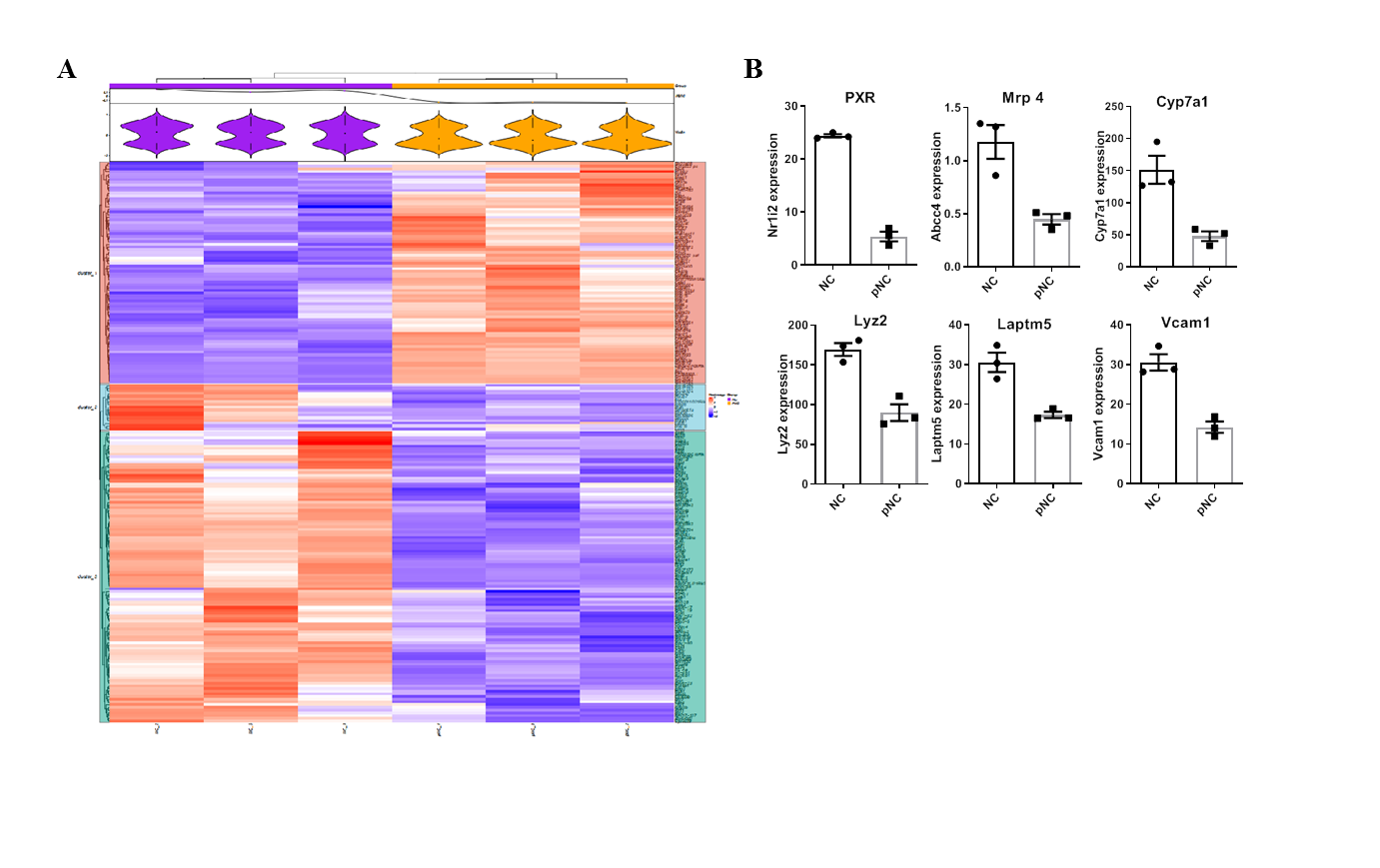

Supplement: Supplementary file 6 — Supplementary Fig. 5 Significantly differentially expressed genes (SDEGs) between the WT and PXR-null groups. [file 41419_2021_3768_MOESM6_ESM.tif]
