# Supplementary material for: Prevention of D-GalN/LPS-induced ALI by 18β-glycyrrhetinic acid through PXR-mediated inhibition of autophagy degradation
Source: Cell Death Dis. 2021 May 13;12(5):480. doi: 10.1038/s41419-021-03768-8 (PMC8119493; doi:10.1038/s41419-021-03768-8)
Supplement: Supplementary file 7 — Supplementary Table 1. [file 41419_2021_3768_MOESM7_ESM.docx]

| **Primer name** | **Sequence (5'→3')** |
| --- | --- |
| r-Vamp8- F | GTGAGGTGGAGGGAGTCAAG |
| r-Vamp8- R | TCTTCCACCAGAACTTCCGG |
| r-Stx17-F | TGACAGCATTGCAGACCATG |
| r-Stx17-R | ATTCCTGCCACTTTGAAGCC |
| r-Tpp1-F | ACTCCATCTGTGCTTCGTCA |
| r- Tpp1-R | TGCTTTCCAACCACTCTTGC |
| r-Cst B-F | CAAGCCTTCCTTTCACCCAC |
| r-Cst B-R | CACTGTTCCCGTGCATCAAA |
| r-PXR-F | CGATTTGCCCTCACCCTGAAGG |
| r-PXR-R | CGTCCGTGCTGCTGAATAACTCC |
| r-Mrp2-F | GGAGCTGGTTGGAAACTTGG |
| r-Mrp2-R | TTGGTCTCTGCTTCTGACGT |
| r-Mrp3-F | GAGAACCTTCACCTCCAGCT |
| r-Mrp3-R | TGGAGCTCACGAACATCAGT |
| r-Mdr1b-F | ACCAGTTCATTCGCTCCTGA |
| r-Mdr1b-R | GGTCGGGTGGGATAGTTGAA |
| r-Gapdh-F | TTCCAGGAGCGAGATCCCGCTAAC |
| r- Gapdh -R | CATGAGCCCTTCCACGATGCCAAAG |
| m-Tpp1-F | GGCAAATGCACTTACAACCCT |
| m- Tpp1-R | CTCCGAGAGTCTTTCCAGGT |
| m-Cst B-F | TTGCGTTCGGTGAGGACATAG |
| m-Cst B-R | GCAGGAGCCCTGGTCTCTA |
| m-Cst E-F | TGTATTCGAGTGTCAATGAACCC |
| m-Cst E-R | GGCTGGTGCAGTACACAGAAG |
| m-Rab7b-F | TCGAGGAATACCAGACCACAC |
| m- Rab7b -R | ACAGCCATCGGAACCTTTGTA |
| m-Rab3il-F | GAAGAGTGTGAACGGCTTTGC |
| m- Rab3il -R | GCTTCCCGAACCATCTTGTGA |
| m-PXR-F | GCGCGGAGAAGACGGCAGCATC |
| m-PXR-R | CCCAGGTTCCCGTTTCCGTGTC |
| m-Mrp2-F | GTGTGGATTCCCTTGGGCTTT |
| m-Mrp2-R | CACAACGAACACCTGCTTGG |
| m-Mdr1b-F | CTGTTGGCGTATTTGGGATGT |
| m-Mdr1b-R | CAGCATCAAGAGGGGAAGTAATG |
| m-Gapdh-F | TCTCCTGCGACTTCAACA |
| m- Gapdh -R | TGGTCCAGGGTTTCTTACT |

**Supplementary table 1 The information of PCR primers**
