# Supplementary material for: Prevention of D-GalN/LPS-induced ALI by 18β-glycyrrhetinic acid through PXR-mediated inhibition of autophagy degradation
Source: Cell Death Dis. 2021 May 13;12(5):480. doi: 10.1038/s41419-021-03768-8 (PMC8119493; doi:10.1038/s41419-021-03768-8)
Supplement: Supplementary file 8 — Supplementary Table 2. [file 41419_2021_3768_MOESM8_ESM.docx]

**Supplementary table 2 The information of antibodies**

| **Antibody** | **Brand** | **Code NO.** | **WB** |
| --- | --- | --- | --- |
| Autophagy Antibody Sampler Kit | cst | #4445 | 1:1000 |
| P62 | MBL | PM045 | 1:1000 |
| Caspase-3 | cst | #9662 | 1:1000 |
| Parp1 | Proteintech | 13371-1-AP | 1:1000 |
| Vamp8 | abcam | ab76021 | 1:10000 |
| Stx17 | Proteintech | 17815-1-AP | 1:1000 |
| Pxr | abcam | ab192579 | 1:500 |
| p-gp | abcam | ab170904 | 1:2500 |
| Gapdh | abcam | ab9485 | 1:2500 |
| beta-Actin | Proteintech | 66009-1-Ig | 1:20000 |
