# Supplementary material for: Prevention of D-GalN/LPS-induced ALI by 18β-glycyrrhetinic acid through PXR-mediated inhibition of autophagy degradation
Source: Cell Death Dis. 2021 May 13;12(5):480. doi: 10.1038/s41419-021-03768-8 (PMC8119493; doi:10.1038/s41419-021-03768-8)
Supplement: Supplementary file 9 — Supplementary Table 3. [file 41419_2021_3768_MOESM9_ESM.docx]

**Supplementary table 3 Gene ontology analysis and upstream analysis of SDEGs**

| **Clusters of SDEGs** | |
| --- | --- |
| Gene ontology | SDEGs |
| Autophagy | Cste, Rab7b, Cebpb, Rab3il1 |
| Inflammation | Ccl6, Ccr5, Cd74, Ifi203, Mmp9, Mmp12, Mx1, Nfam1, Tifab, Tlr13, Tlr3 |
| Metabolism and transport | Cyp17a1, Cyp2b10, Slc16a3, Slc25a30, Slc38a1, Slc40a1, Slc43a2 |
| **Upstream analysis of SDEGs** | |
| Genes (promoter region is bound by Pxr in the liver of mice (ChIP-seq)) | |
| Ccl6, Cyp17a1, Cyp2b10, Exo3l2, Foxq1, Gas1, Gstm3, Hamp2, Scara5, Slc16a3, Slc25a30, Slc38a1, Slc40a1, Slc43a2, Smpd3 | |
